# Isoform-Specific Na,K-ATPase Alterations Precede Disuse-Induced Atrophy of Rat Soleus Muscle

**DOI:** 10.1155/2015/720172

**Published:** 2015-01-13

**Authors:** Violetta V. Kravtsova, Vladimir V. Matchkov, Elena V. Bouzinova, Alexander N. Vasiliev, Irina A. Razgovorova, Judith A. Heiny, Igor I. Krivoi

**Affiliations:** ^1^St. Petersburg State University, 7/9 University emb., St. Petersburg 199034, Russia; ^2^Aarhus University, Ole Worms Alle bygn. 4, 1163, C 8000 Aarhus, Denmark; ^3^University of Cincinnati College of Medicine, Cincinnati, OH 45267, USA

## Abstract

This study examines the isoform-specific effects of short-term hindlimb suspension (HS) on the Na,K-ATPase in rat soleus muscle. Rats were exposed to 24–72 h of HS and we analyzed the consequences on soleus muscle mass and contractile parameters; excitability and the resting membrane potential (RMP) of muscle fibers; the electrogenic activity, protein, and mRNA content of the *α*1 and *α*2 Na,K-ATPase; the functional activity and plasma membrane localization of the *α*2 Na,K-ATPase. Our results indicate that 24–72 h of HS specifically decreases the electrogenic activity of the Na,K-ATPase *α*2 isozyme and the RMP of soleus muscle fibers. This decrease occurs prior to muscle atrophy or any change in contractile parameters. The *α*2 mRNA and protein content increased after 24 h of HS and returned to initial levels at 72 h; however, even the increased content was not able to restore *α*2 enzyme activity in the disused soleus muscle. There was no change in the membrane localization of *α*2 Na,K-ATPase. The *α*1 Na,K-ATPase electrogenic activity, protein and mRNA content did not change. Our findings suggest that skeletal muscle use is absolutely required for *α*2 Na,K-ATPase transport activity and provide the first evidence that Na,K-ATPase alterations precede HS-induced muscle atrophy.

## 1. Introduction

Skeletal muscle activity is absolutely required to maintain both muscle mass and muscle function. A variety of conditions are known to produce muscle atrophy, including unloading and reduced neuronal activity (disuse) [1, 2]. Recent studies indicate that the regulatory mechanisms which operate at early stages of skeletal muscle atrophy differ from those during chronic muscle inactivity. Investigations into the regulatory processes and interactions that occur early in the development of muscle atrophy will be needed to discover the molecular mechanisms which remodel skeletal muscles during adaptations to disuse [1].

Maintaining the necessary level of the resting membrane potential (RMP) in skeletal muscles is obligatory for successful functioning of the neuromuscular system [3, 4]. It is well known that hindlimb suspension (HS), a widely used model of disuse and hypogravity, leads to progressive atrophy of postural skeletal muscle [1, 5, 6]. HS lasting three days or longer induces dramatic remodeling events in the rat soleus muscle that include slow-to-fast shift in myosin heavy chain expression pattern, changes in the neuromuscular junction, changes in ion channels expression as well as depolarization of the RMP [7–11]. This depolarization has been shown to result from a decrease in the electrogenic activity of the Na,K-ATPase [10, 11].

The Na,K-ATPase is a P-type ATPase which catalyzes the active transport of K^+^ into and Na^+^ out of the cell, thereby maintaining the steep Na^+^ and K^+^ gradients that provide electrical excitability and the driving force for many other transport processes. The Na,K-ATPase is composed of *α*-catalytic and *β*-glycoprotein subunits. Four isoforms of the *α* subunit are known to exist in tissues of vertebrates. It is generally accepted that the ubiquitous *α*1 isoform plays the main “house-keeping” role while the other isoforms are expressed in a cell- and tissue-specific manner and possesses additional regulatory functions [12–14].

The Na,K-ATPase is critically important for excitability, electrogenesis and contractility of skeletal muscle [15–17]. The largest pool of the Na,K-ATPase in a vertebrate's body is located in skeletal muscles where *α*1 and *α*2 isoforms are coexpressed [18]. The mechanisms by which the *α*1 and *α*2 isoforms are regulated in response to changes in skeletal muscle activity remain to be elucidated [17, 19–23]. Moreover the Na,K-ATPase isoform-specific changes during muscle inactivity or disuse have not been studied in detail. The effect of short-term HS on the Na,K-ATPase isoforms is not known, although this basic information on a protein as vital as the Na,K-ATPase is expected to advance our understanding of the cellular and molecular mechanisms responsible for disuse-induced muscle atrophy.

This work examines the role of the Na,K-ATPase *α* isozymes in the early adaptations of skeletal muscle to disuse. We subjected rats to short periods of HS (24 or 72 h) and measured the consequences on soleus muscle mass and contractile parameters; muscle electrogenesis and excitability; the electrogenic activity of the *α*1 and *α*2 Na,K-ATPase isozymes; their protein and mRNA content; the plasma membrane localization of the *α*2 Na,K-ATPase and its transport capacity.

Our findings suggest that short-term HS alters the Na,K-ATPase of rat soleus muscle in an isoform-specific manner and indicate that *α*2 Na,K-ATPase alterations precede disuse-induced muscle atrophy.

## 2. Materials and Methods

### 2.1. Animals

Experiments were performed on male Wistar rats (180–230 g). The animals were subjected to HS individually in custom cages for 24 or 72 h, as described [5]. Control animals were not suspended. After HS, all rats were anesthetized by ether and euthanized by cervical dislocation, and soleus muscles were removed.

### 2.2. Ethics Statement

This study was carried out in accordance with the recommendations for the Guide for the Care and Use of Laboratory Animals of the National Institutes of Health. The protocol was approved by the Ethics Committee of St. Petersburg State University and the National Ministry of Health of the Russian Federation. All surgery was performed under anesthesia (ether), and all efforts were made to minimize suffering.

### 2.3. Membrane Potential Recording

The isolated muscle with nerve stump was placed in a chamber and continuously perfused with a physiological solution containing (mM): NaCl, 137; KCl, 5; CaCl_2_, 2; MgCl_2_, 2; NaHCO_3_, 24; NaH_2_PO_4_, 1; glucose, 11; pH 7.4. The solution was continuously bubbled with 95% O_2_ and 5% CO_2_ and maintained at 28°C. The RMPs of muscle fibers were recorded from extrajunctional membrane regions using intracellular microelectrodes, as described previously [24, 25]. RMPs were recorded from 25 to 35 different fibers within each muscle, over a total recording time of about 5–10 min. The entire protocol was repeated in muscles from different animals.

### 2.4. Measurement of Na,K-ATPase Electrogenic Activity in Intact Muscle

Na,K-ATPase transport activity was determined in intact muscle fibers by measuring the ouabain-sensitive change in RMP. This change is generated by electrogenic Na,K-ATPase transport and is a sensitive, real-time assay of Na,K-ATPase activity in intact skeletal muscle cells. The method is based on the more than 100-fold difference in affinities of the rodent *α*1 and *α*2 Na,K-ATPase isoforms for ouabain [25–27]. The electrogenic contribution of the *α*2 isozyme was computed as the difference in mean RMP before and after 30 min incubation in 1 *μ*M ouabain. The electrogenic contribution of the *α*1 isozyme was estimated as the difference between the RMP in 1 *μ*M ouabain and after 30 min incubation with 500 *μ*M ouabain. Total Na,K-ATPase electrogenic activity was determined as the difference between the RMPs prior to any ouabain addition and after 30 min action of 500 *μ*M ouabain.

### 2.5. Contraction Measurements

Isolated muscles were mounted in a chamber; one tendon was fixed, while the other end was connected to the isometric FT03C force transducer (Grass Instrument Co., Quincy, Massachusetts, United States). Electrical direct stimulation (single pulses of 1 ms) was performed with two silver chloride electrodes using Isostim A320 (WPI, USA) stimulator. Optimum muscle length was determined by twitch tension from supramaximal stimulation. The electrodes were placed onto the nerveless part of the muscle. Peak twitch tension, time to peak (10–90% of the peak tension), and half-relaxation time were recorded.

### 2.6. Western Blot Analysis

Isolated soleus muscles were lysed in lysis buffer (in mM: Tris-HCl 10, sucrose 250, EDTA 1, EGTA 1, Triton X-100 2%, pH 7.4; and 1 tablet protease inhibitor per 10 mL). The homogenate was centrifuged at 10,000 g and the supernatant was collected. The protein-containing supernatant was adjusted with 1 mol/L DTT and 2x trisglycine SDS sample buffer (Invitrogen, Denmark) with an approximate ratio of 10 : 3 : 3, respectively. Ten *μ*g of protein was loaded to gels. Proteins were separated on 10% trisglycine gels and electrotransferred onto nitrocellulose membranes, which were then blocked by incubating in 5% nonfat dry milk in PBS (in mM: NaCl 137, KCl 2.7, Na_2_HPO_4_ 8.2, KH_2_PO_4_ 1.8, at pH 7.4) with 0.5% vol/vol tween 20 (PBS-T). The membranes were incubated with primary antibodies (*α*1 isoform of the Na,K-pump antibody (1 : 2000, Santa Cruz Biotechnology Inc., USA) and *α*2 isoform of the Na,K-pump antibody (1 : 2000, Millipore, USA) overnight at 5°C in PBS-T. After washing, the membranes were incubated with horseradish-peroxidase- (HRP-) conjugated secondary antibody (1 : 4000; Dako, Denmark) for 1 hour in PBS-T. Excess antibody was removed by extensive washing, and bound antibody was detected by an enhanced chemiluminiscence kit (ECL, Amersham, UK). Membranes were then stripped for antibodies and stained for pan-actin (Cell Signaling Technology Inc., USA) with a HRP-conjugated secondary antibody. Detected protein was quantified as a ratio to pan-actin intensity measured for the same probe, using ImageJ software (NIH, USA).

### 2.7. Quantitative Polymerase Chain Reaction (PCR)

Isolated soleus muscle segments were disrupted in Tissue Lyser (Qiagen, VWR, Denmark). RNA isolation was carried out with Qiagen Mini kit (Qiagen, VWR, Denmark) in a QIAcube robotic workstation for automated purification of RNA (Qiagen, VWR, Denmark). PCR was performed to assess the expression of specific RNAs. The reaction was executed with reverse transcriptase III (Invitrogen, Denmark) and superase (Ambion Ltd., UK) for deactivation of RNAse and DNAse. The standard primer sets for quantitative PCR analyses for *α*1 and *α*2 isoforms of the Na,K-pump were obtained from Applied Biosystems (Denmark). Quantitative PCR was carried out on MX3000P (Stratagene, USA) using Taqman probe (FAM) technology. Gene expression was normalized to GAPDH and transferrin receptor (average Ct value) levels and presented by a ΔCt value. Comparison of gene expression was derived by subtracting control ΔCt (an averaged ΔCt for the muscles which were not exposed to HS) from sample ΔCt, producing ΔΔCt. Relative gene expression was calculated as 1/(2^ΔΔCt^), thereby standardizing to control muscle.

### 2.8. Confocal Microscopy and Membrane Localization

Ouabain at 1 *μ*M selectively inhibits the rodent *α*2 Na,K-ATPase without effect on the *α*1 isoform, as shown previously [26, 27]. For selective imaging of the Na,K-ATPase *α*2 isoform, a freshly isolated m. soleus was incubated for 15 min with physiological saline containing fluorescent-labeled specific ligand of the Na,K-ATPase (bodipy-conjugated ouabain, 1 *μ*M). Superficial regions of the muscle were imaged with a ×40, 1.3 NA objective using a Leica TCS SP5 confocal system configured for viewing of bodipy fluorescence, as was described previously [25, 28].

### 2.9. Immunofluorescent Detection of the Na,K-ATPase *α*2 Isozyme

Skeletal muscles from control and HS rats fixed in 4% formaldehyde were washed with the phosphate-buffered salt solution (PBS) containing (in mmol/L): NaCl 137, KCl 2.7, Na_2_HPO_4_ 8.2, KH_2_PO_4_ 1.8, at pH 7.4. Then, unreacted fixative was quenched with 25 mmol/L glycine in PBS for 15 minutes. Muscle fibers were washed 3 times with PBS, permeabilized with 0.1% Triton-X in PBS for 1 h and incubated with primary *α*2 isoform of the Na,K-pump antibody (1 : 100, Millipore, USA) overnight at 5°C. After 3 wash, muscles were incubated in the dark with Alexa-488 fluorescent conjugated secondary antibody for 1 h at room temperature (1 : 2000; Invitrogen, USA). After washing the preparation was transferred to the confocal microscope (LSM-5 Pascal Exciter, Zeiss, Germany). The emission signal at 530 nm (after excitation at 488 nm) was stored on the computer for later analyses of fluorescence intensity using ImageJ (NIH, USA).

### 2.10. Cross Sectional Area Analysis

Contrast images of transverse section of formaldehyde fixed soleus muscle were taken microscopically for following analyses of cross sectional area of individual muscle fibers. The analysis was done using ImageJ (NIH, USA).

### 2.11. Materials

Bodipy-conjugated ouabain was from Invitrogen. Ouabain, nicotine ((−)nicotine hydrogen tartrate) and other chemicals were from Sigma-Aldrich.

Data are given as the mean ± SEM. Statistical significance of the difference between means was evaluated using a Student's *t*-test (ORIGIN 6.1. software) and one way ANOVA (ORIGIN Pro 8 software).

## 3. Results

### 3.1. Hindlimb Suspension Alters Na,K-ATPase Electrogenic Activity and Electrogenesis prior to Muscle Atrophy or Any Change in Contractility

**Figure 1:**
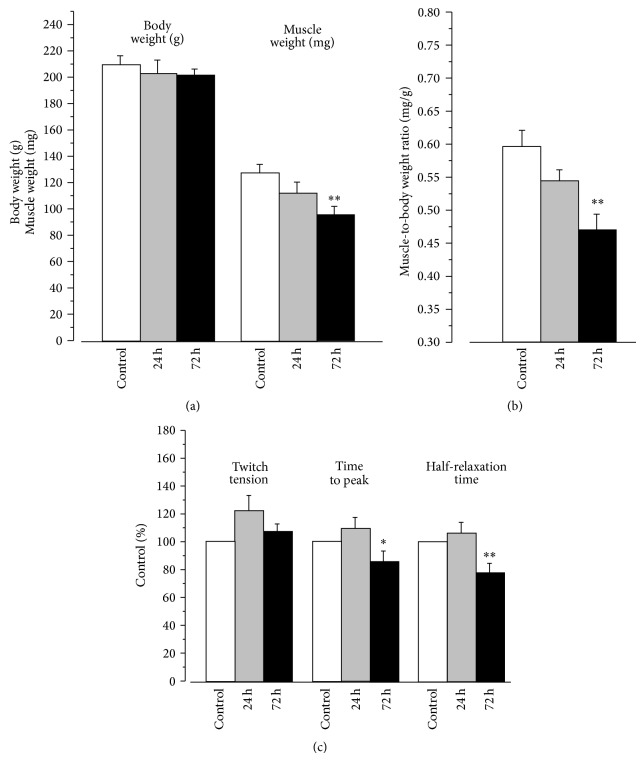
Effects of 24–72 h of hindlimb suspension on body and muscle weights (a), muscle-to-body weight ratio (b), and parameters (c) of rat soleus twitch tensions. ^*^ *P* < 0.05; ^**^ *P* < 0.01 compared to control.

The body and soleus muscle weights of control rats were 209.8 ± 6.5 g (40 rats) and 127.3 ± 6.4 mg (40 pairs of muscles), respectively. The body weight was not changed during HS while the soleus muscle weight significantly (*P* < 0.01) decreased to 96.0 ± 5.9 mg (22 pairs of muscles) (Figure 1(a)). As a useful index of muscle atrophy, we determined changes of the muscle mass expressed as soleus muscle weight (mg) normalized to the whole body weight (g). In control soleus muscle the muscle-to-body weight ratio was 0.60 ± 0.02 mg/g (40 rats, 40 pairs of muscles) similar to published values [29]. Only a slight and non-significant decrease of this ratio was observed after 24 h of HS. However this ratio significantly (*P* < 0.01) decreased to 0.47 ± 0.02 mg/g (22 rats, 22 pairs of muscles) after 72 h of HS (Figure 1(b)), corresponding well with the 11% decrease in mean cross sectional area of individual soleus muscle fibers (from 1687 ± 22 *μ*m^2^, 502 fibers, in control down to 1511 ± 21 *μ*m^2^, 475 fibers, after HS, *P* < 0.01). Mean cross sectional area after 24 h of HS was not determined.

24 h of HS did not alter twitch parameters compared to control (23 and 18 muscles, resp.). Whereas, prolonged HS of 72 h (8 muscles) decreased time-to-peak and half-relaxation time by 13% (*P* < 0.05) and 21% (*P* < 0.01), respectively (Figure 1(c)).

**Figure 2:**
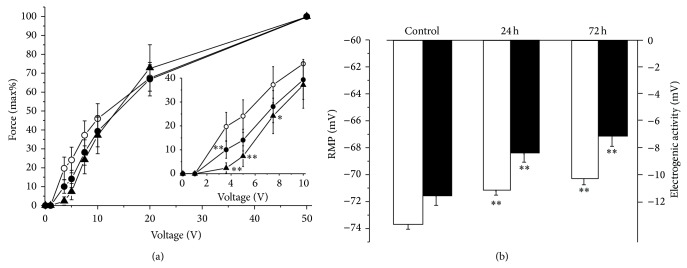
Hindlimb suspension decreases excitability and alters electrogenesis of rat soleus muscle. (a) force-voltage relationships of twitch tensions in control soleus muscles (open circles, *n* = 18), after 24 h (closed circles, *n* = 15) and 72 h (triangles, *n* = 8) of HS. Force-voltage relationships were determined using 1 ms stimuli (direct stimulation) of increasing voltage to elicit maximum force of twitch tension. % to force obtained at supramaximal stimulation is shown. Insert-expanded curve for voltages below 10 mV; points that significantly differ from corresponding control points are marked by asterix. (b) The resting membrane potential (white columns) and total electrogenic activity of the Na,K-ATPase (black columns) in control soleus muscles and after 24 h and 72 h of HS. ^*^ *P* < 0.05; ^**^ *P* < 0.01 compared to respective control.

24–72 h of HS significantly shifted the initial part of the force-voltage relationship to higher voltages, suggesting decreased excitability (Figure 2(a)). Over the same period, 24 h of HS depolarized the RMPs of extrajunctional membrane regions from −73.7 ± 0.4 mV (10 muscles, 211 fibers) in control muscles to −71.2 ± 0.3 mV (12 muscles, 269 fibers), and this value remained unchanged (−70.2 ± 0.6 mV; 8 muscles, 126 fibers) up to 72 h of HS (Figure 2(b)).

Total electrogenic activity of the Na,K-ATPase was determined by measuring the ouabain-sensitive change in RMP (see Section 2). In the extrajunctional region of control soleus muscles, total electrogenic activity by the Na,K-ATPase contributes −11.6 ± 0.7 mV (6 muscles) to the RMP. After 24 h of HS, the electrogenic potential contributed by the Na,K-ATPase decreased to −8.5 ± 0.6 mV (6 muscles, *P* < 0.01) and this value remained unchanged up to 72 h of HS (Figure 2(b)). Therefore, the HS-induced membrane depolarization and lowered muscle excitability are due to decrease in the Na,K-ATPase electrogenic activity.

These data indicate that the decrease in Na,K-ATPase electrogenic activity, RMP and excitability all precede the HS-induced muscle atrophy and changes in contractility.

### 3.2. Hindlimb Suspension Specifically Alters *α*2 Na,K-ATPase

**Figure 3:**
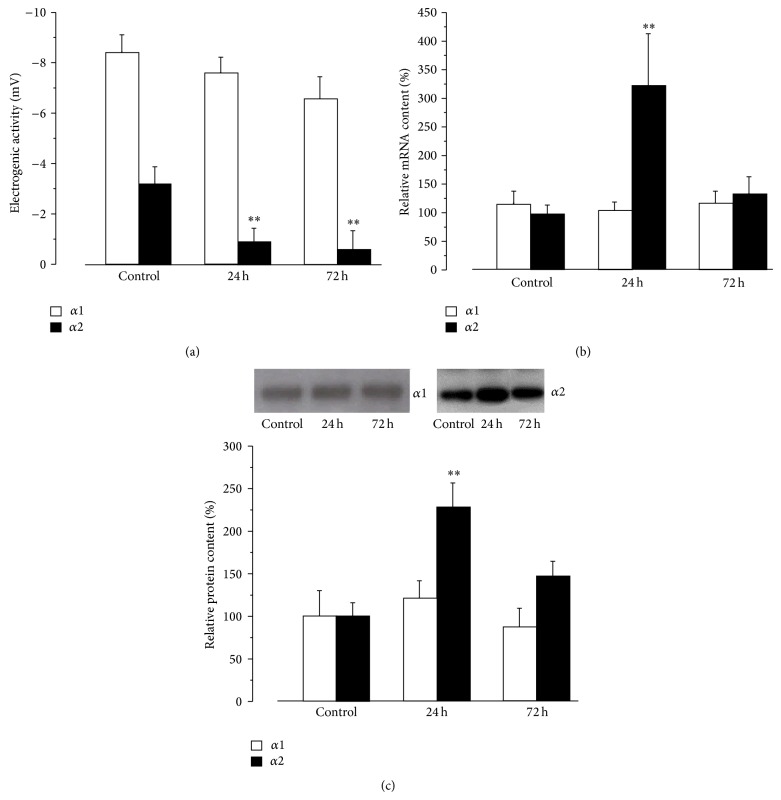
24–72 h of hindlimb suspension specifically alters the electrogenic activity (a) and the relative mRNA (b) and protein (c) contents of the *α*2 Na,K-ATPase in rat soleus muscle. *α*1 and *α*2 Na,K-ATPase, white and black columns, respectively. (a) Na,K-ATPase *α*1 and *α*2 electrogenic activity computed as the difference in resting potential measured before and after blockade of the Na,K-ATPase isozymes by ouabain. (b) and (c) data normalized to the average level of expression under control conditions. Upper panels (c) show representative immunoblots. Columns show mean data from 5 to 10 different muscle samples (panel (b)) and 10 different muscle samples (panel (c)). ^**^ *P* < 0.01 compare to control.

In control muscles, electrogenic transport by the *α*1 and *α*2 Na,K-ATPase isozymes contributes −8.4 ± 0.7 mV and −3.2 ± 0.7 mV (6 muscles), respectively, to the RMP. HS alters these contributions in an isoform-specific manner. After 24 h of HS, the electrogenic potential contributed by *α*1 isozyme remains unchanged, while the contribution of *α*2 isozyme decreased dramatically to only −0.9 ± 0.6 mV (6 muscles; *P* < 0.01) (Figure 3(a)). The lower activity of *α*2 isozyme remained for up to 72 h of HS while the contribution of *α*1 isozyme was not significantly different from control value (Figure 3(a)). Therefore, the HS-induced membrane depolarization is due to a specific decrease in the activity of the *α*2 isozyme.

After 24 h of HS both *α*2 Na,K-ATPase mRNA and protein content increased, while *α*1 protein and mRNA content did not change (Figures 3(b) and 3(c)). These data suggest that the HS-induced decrease in *α*2 Na,K-ATPase activity is due to a decrease in enzyme activity and not to altered mRNA or protein content. Moreover, even the increased *α*2 Na,K-ATPase mRNA and protein content at 24 h of HS cannot counteract the sustained inhibition of *α*2 isozyme electrogenic activity (Figure 3(a)). The increase in *α*2 mRNA and protein content in response to disuse was transient. After 72 h of HS, the mRNA and protein content of both *α*1 and *α*2 Na,K-ATPase were not different from control (Figures 3(b) and 3(c)).

### 3.3. Hindlimb Suspension Does Not Alter the Functional Activity or Membrane Localization of the *α*2 Na,K-ATPase

As a confirmation that *α*2 Na,K-ATPase remains in the membrane and is functional after HS, we tested whether the Na,K-ATPase *α*2 isozyme is able to be stimulated by nanomolar concentrations of nicotinic acetylcholine receptor (nAChR) agonists, an effect shown previously [24, 25].

**Figure 4:**
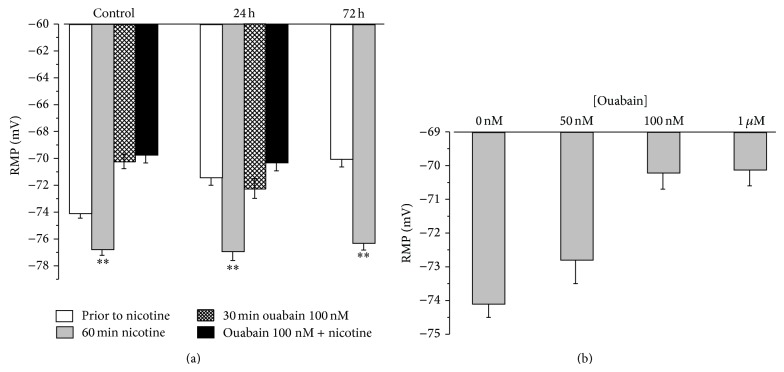
Changes in the resting membrane potential induced by 100 nM nicotine in rat soleus muscle in control, after 24 and 72 h of hindlimb suspensions. (a) RMPs were measured in the same muscles prior to (white columns) and after 60 min of nicotine application (grey columns); 12 muscles, control, 4 muscles, after 24 h and 8 muscles after 72 h of HS; in separate muscles RMPs were measured after 30 min of 100 nM ouabain application (cross hatched columns) and in the presence of ouabain after 60 min of nicotine application (black columns). 4 muscles, control, 4 muscles, after 24 h of HS. ^**^ *P* < 0.01 compared to RMPs before nicotine's addition. (b) the effect of ouabain (30 min incubation) on the RMPs of control rat soleus muscles. Columns show mean data from 4 to 6 different muscles.

Upon 60 min exposure of muscles to 100 nM nicotine, a small but significant (*P* < 0.01) depolarization was detected (not shown), as expected if nicotine initially opens a small number of nAChRs. This depolarization was followed by sustained hyperpolarization of 2.7 ± 0.6 mV (*P* < 0.01; 12 muscles) (Figure 4(a)). After 24 or 72 h of HS, the RMPs depolarized as indicated above; however the stimulation of Na,K-ATPase activity by nicotine remained (Figure 4(a)), indicating the continued presence of functional Na,K-ATPase in the sarcolemma.

To verify whether the nicotine-induced hyperpolarization is actually related to activation of Na,K-ATPase *α*2 isozyme, additional experiments with ouabain were performed. The rodent *α*1 isozyme is 100-fold less sensitive to ouabain binding, and 1 *μ*M ouabain completely inhibits the *α*2 isozyme without effect on the *α*1 isozyme [24, 26, 27]. In control muscles, ouabain (50 nM, 100 nM or 1 *μ*M; 30 min incubation) depolarized muscle fibers in a dose-dependent manner to ~−70 mV, as expected (Figure 4(b)). The nicotine-induced hyperpolarization was absent in the presence of 100 nM ouabain (30 min preincubation), confirming that it results from stimulated electrogenic transport by the Na,K-ATPase *α*2 isozyme (Figure 4(a)). 24 h of HS depolarized RMPs to the same level as in the presence of 100 nM to 1 *μ*M ouabain in control muscles, suggesting inhibition of the Na,K-ATPase *α*2 isozyme after HS (Figure 4(b)). As in control muscles, 100 nM ouabain prevents the nicotine-induced hyperpolarization (Figure 4(a)).

These data indicate that the Na,K-ATPase *α*2 isozyme, even after being inhibited by HS, remains capable of dynamically increasing its transport activity in response to nanomolar nAChR agonist.

**Figure 5:**
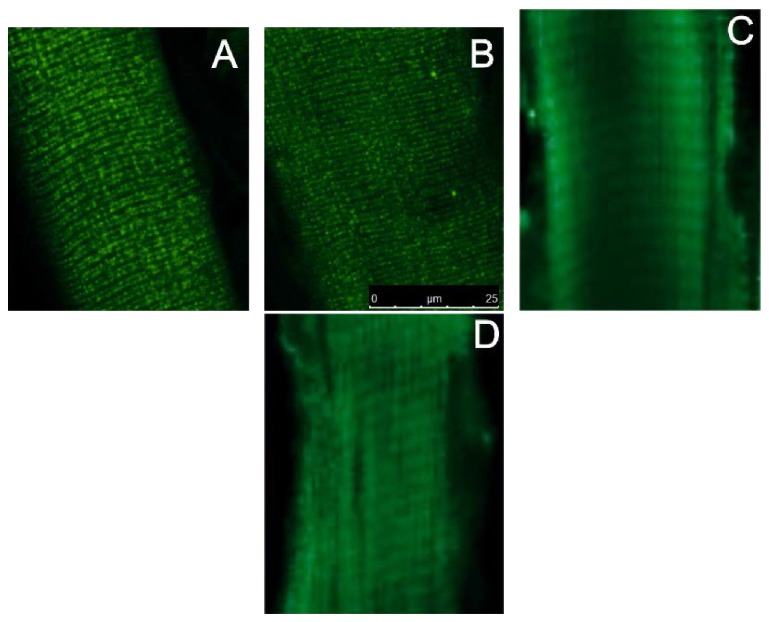
Hindlimb suspension does not alter the membrane localization of *α*2 Na,K-ATPase. A rat soleus muscle was labeled with bodipy-conjugated ouabain (1 *μ*M) ((A), (B)) or with specific antibodies ((C), (D)) to label the *α*2 Na,K-ATPase. (A), (C) control; (B) after 24 h HS and (D) after 72 h of HS. Scale bars: 25 *μ*m.

It is also possible that HS may alter the localization of *α*2 Na,K-ATPase in the sarcolemma and thereby alter its electrogenic activity. We investigated this possibility by imaging the intact m. soleus labeled with a fluorescent, specific ligand of the Na,K-ATPase (bodipy-conjugated ouabain, 1 *μ*M) [25, 28]. The *α*2 Na,K-ATPase isozyme is present on the sarcolemma and transverse tubule membranes [30]. Consistent with this observation, the fluorescent ouabain signal is detected as double rows of label with a repeat pattern of two per sarcomere, as expected from the dual transverse-tubule openings at the A-I junctions of mammalian muscle [25, 28] (Figure 5(A)). 24 h of HS did not alter the extrajunctional localization of the Na,K-ATPase *α*2 isozyme (Figure 5(B)). Muscles labeled with *α*2 Na,K-ATPase antibody demonstrate the same result after 72 h of HS (Figures 5(C) and 5(D)).

Taken together, these data suggest that the inhibition of the *α*2 Na,K-ATPase electrogenic activity induced by short-term HS cannot be explained by altered functional capacity and/or by altered localization in the m. soleus sarcolemma.

## 4. Discussion

Investigations into the molecular mechanisms which trigger downstream signaling events leading to muscle atrophy are of great importance [1, 2, 6]. The Na,K-ATPase is the most abundant protein in skeletal muscle and is one of the marker enzymes that sense enhanced mechanical activity. The mechanisms by which the Na,K-ATPase is regulated in response to skeletal muscle activity are incompletely understood. A variety of processes have been suggested to regulate Na,K-ATPase activity or content, including: increased Na^+^ entry [19, 21, 22], an enhanced level of cytosolic Ca^2+^ [23, 31], changes in ion affinity and maximal enzyme activity [22], translocation of Na,K-ATPase subunits [19, 20, 32], AMPK activation [33], oxidative stress (glutathionylation) [34] and other factors.

The cellular mechanisms of disuse-induced Na,K-ATPase deregulation are only starting to be elucidated. In the present study, we demonstrate for the first time that the Na,K-ATPase is altered during earlier stages of rat soleus disuse, which precede muscle atrophy, and that the *α*2 Na,K-ATPase isozyme is specifically targeted.

The *α*1 and *α*2 isoforms of Na,K-ATPase are coexpressed in the skeletal muscles [18]. The *α*2 isoform is the major *α* subunit and displays marked adaptability compared to the *α*1 isoform [14, 16, 17]. Recent studies of the role of the *α*2 Na,K-ATPase suggest that the *α*2 isoform is essential for the contractile function of skeletal muscle and that its specific transport activity can be acutely regulated by muscle activity [25, 28, 35–37].

Many other studies have established that the total content of Na,K-ATPase mRNA and protein is regulated by muscle activity over a regulatory range of about 2-fold (reviewed in [16, 17]). Chronic training of various intensities and duration upregulates the total content of Na,K-ATPase, and disuse induced by immobilization, tenotomy, or immobilization downregulates Na,K-ATPase content. Several studies have suggested that the content of *α*2 subunit in skeletal muscle is relatively more sensitive to chronic changes in muscle activity than *α*1, but the reported results are somewhat variable (summarized in [17]; Table 2).

In this study, we examined whether *α*2 Na,K-ATPase function or content is conversely regulated by muscle inactivity. HS, a common experimental model of muscle disuse, leads to atrophy associated with well-characterized phenotypic changes in fiber type, protein expression, and function [1, 2, 6]. Membrane depolarization is an early event associated with this model [10]. Our present data indicate that the decrease in the RMP and excitability of rat soleus muscle results from reduced Na,K-ATPase electrogenic activity which precedes the HS-induced muscle atrophy and changes in contractility. The muscle membrane depolarization occurs as early as 24 h of HS. Importantly, it is isoform-specific and results from a specific decrease in the electrogenic activity of the Na,K-ATPase *α*2 isozyme. The loss of *α*2 Na,K-ATPase activity occurs prior to any detectable change in muscle mass or contractility, and despite an increase in *α*2 mRNA and protein content. The increases in *α*2 mRNA and protein content are transient; both return to near baseline levels at 72 h of HS. Importantly, the *α*2 Na,K-ATPase isozyme remains capable of stimulation by cholinergic regulatory mechanisms and its localization in the membrane is not altered during 24–72 h of HS.

Other studies indicate that animals show an initial, transient stress response to HS. Adrenal hypertrophy and corticosterone levels increase and peak within the first few days of HS, then decline as the animal adapts [38]. A recent study reports that corticosterone levels increased slightly after 6 h of HS, then returned to control levels during the subsequent 12–72 h of HS [39]. Because prolonged treatment (14 d) of rats to Dexamethasone, a potent synthetic corticosteroid, increases *α*2 Na,K-ATPase protein and mRNA abundance in skeletal muscles [40], we considered whether an early stress response might occur in our animals and might explain the results. However, our results show clearly that the acute, HS-induced decrease in *α*2 Na,K-ATPase electrogenic activity is a functional change in enzyme activity, and is not associated with reduced *α*2 mRNA or protein content. Indeed, *α*2 mRNA and protein increase transiently at 24 h, then return to control levels after 72 h of HS. Therefore, the short-term downregulation of *α*2 Na,K-ATPase electrogenic activity by HS cannot be explained by a stress response. Moreover, even the increased *α*2 Na,K-ATPase mRNA and protein content after 24 h of HS cannot counteract the sustained inhibition of *α*2 isozyme activity. Collectively, these findings suggest that the activity of pre-existing *α*2 Na,K-ATPase is regulated by muscle use.

The transient increase in *α*2 mRNA and protein content seen at 24 h, and its return to control levels by 72 h of HS, may reflect the complex dynamics of muscle gene adaptations to disuse. In particular, skeletal muscles at fatigue show decreased Na,K-ATPase activity that is inversely correlated with increased *α*2 mRNA expression, suggesting a possible signal-transduction role for depressed Na,K-ATPase activity on *α* isoform gene expression [41]. Such increase in *α*2 mRNA expression at fatigue may be a compensatory response to preserve muscle function.

The mechanism by which the electrogenic activity of *α*2 Na,K-ATPase is downregulated in response to muscle disuse is not known. Our data indicate that *α*2 Na,K-ATPase remains in the membrane and is able to be stimulated by other regulatory pathways including its interaction with the nAChR. This stimulation is realized via functional interaction between the nAChRs and the *α*2 Na,KATPase, as was shown previously [24, 25]. Nanomolar concentrations of acetylcholine remain in the junctional cleft and muscle interstitial spaces for some time following nerve activity [25]. The EMG activity of soleus muscle decreases dramatically immediately following HS and remains lowered for at least up to 3 days of HS [42, 43]. A reduced EMG indicates reduced motor nerve activity, which would lower the level of “residual” interstitial acetylcholine and reduce stimulation of *α*2 Na,K-ATPase by interaction with the nAChRs.

Other factors related to muscle inactivity may be involved in the inhibition of *α*2 Na,K-ATPase functional activity during short-term HS. The regulation of the *α*2 isozyme may also depend on its subunit partners and/or molecular environment, which are not completely known. The submembranous actin-based cytoskeleton has been shown to participate in the regulation of Na,K-ATPase activity, and HS is known to alter the transverse stiffness of rat soleus muscle via non-muscle alpha-actinins [39]. In addition, PKC activity is known to decrease after short HS [29], and this in turn could alter the phosphorylation state of the Na,K-ATPase *α* or FXYD1 subunits [44, 45].

It is well known that calcium ions play an essential role for a diversity of muscle functions [46]. Some data indicate that resting intracellular calcium levels increase in m. soleus fibers even after first days of HS [6, 10, 47] and it was proposed that this might contribute to the activation of calpains, further promoting muscle atrophy [6, 48]. So, the increase in basal Ca^2+^ level might act as a key trigger to downstream signaling events leading to muscle atrophy. The sources and mechanisms of this calcium accumulation remained to be elucidated. At the present time we can only speculate that the basal Ca^2+^ level might increase due to altered Na,Ca-exchange as a result of lowered *α*2 Na,K-ATPase transport activity and Na^+^ accumulation, similarly to “PLasmERosome” model [49].

Together, these findings demonstrate that muscle activity is absolutely required for the acute regulation of *α*2 transport activity. Overall, they support an emerging body of evidence suggesting that the catalytic activity of the *α*2 Na,K-ATPase isozyme in skeletal muscle is exquisitely sensitive to muscle use. Acute increases in muscle activity stimulate *α*2 isozyme transport activity, while acute decreases in muscle activity downregulate its activity. Longer-term changes in the level of muscle use also influence *α*2 mRNA and protein content; the changes in content induced by HS are transient and therefore, whether a change in *α*2 content is observed depends critically on the time point of the measurement.

Our findings further show that isoform-specific changes in Na,K-ATPase expression and function are an early event in the development of disuse atrophy during HS. The significance of these early changes in Na,K-ATPase transport activity, content, and membrane depolarization in the development of disuse atrophy remains to be elucidated.

## Abbreviations

HS: Hindlimb suspension
RMP: Resting membrane potential
nAChR: Nicotinic acetylcholine receptor
EMG: Electromyogram
PKC: Protein kinase C.

## References

[1] Baldwin K. M., Haddad F., Pandorf C. E., Roy R. R., Edgerton V. R. (2013). Alterations in muscle mass and contractile phenotype in response to unloading models: role of transcriptional/pretranslational mechanisms. *Frontiers in Physiology*.

[2] Bodine S. C. (2013). Disuse-induced muscle wasting. *International Journal of Biochemistry and Cell Biology*.

[3] Wood S. J., R. Slater C. (2001). Safety factor at the neuromuscular junction. *Progress in Neurobiology*.

[4] Ruff R. L. (2011). Endplate contributions to the safety factor for neuromuscular transmission. *Muscle & Nerve*.

[5] Morey-Holton E., Globus R. K., Kaplansky A., Durnova G. (2005). The hindlimb unloading rat model: literature overview, technique update and comparison with space flight data. *Advances in Space Biology and Medicine*.

[6] Shenkman B. S., Nemirovskaya T. L. (2008). Calcium-dependent signaling mechanisms and soleus fiber remodeling under gravitational unloading. *Journal of Muscle Research and Cell Motility*.

[7] Mounier Y., Bacou F., Falempin M. (1995). Changes in the neuromuscular junction of rat soleus muscle after hindlimb unweighting. *Basic and Applied Myology*.

[8] Desaphy J.-F., Pierno S., Léoty C., George A. L., De Luca A., Camerino D. C. (2001). Skeletal muscle disuse induces fibre type-dependent enhancement of Na^+^ channel expression. *Brain*.

[9] Pierno S., Desaphy J.-F., Liantonio A. (2002). Change of chloride ion channel conductance is an early event of slow-to-fast fibre type transition during unloading-induced muscle disuse. *Brain*.

[10] Krivoǐ I. I., Kravtsova V. V., Altaeva E. G. (2008). A decrease in the electrogenic contribution of Na,K -ATPase and resting membrane potential as a possible mechanism of Ca^2+^ accumulation in musculus soleus of the rat at short-term gravity unloading. *Biofizika*.

[11] Tyapkina O., Volkov E., Nurullin L. (2009). Resting membrane potential and Na^+^,K^+^-ATPase of rat fast and slow muscles during modeling of hypogravity. *Physiological Research*.

[12] Blanco G., Mercer R. W. (1998). Isozymes of the Na-K-ATPase: heterogeneity in structure, diversity in function. *American Journal of Physiology*.

[13] Geering K. (2008). Functional roles of Na,K-ATPase subunits. *Current Opinion in Nephrology and Hypertension*.

[14] Krivoǐ I. I. (2012). Regulatory function of the Na,K-ATPase alpha 2 isoform. *Biofizika*.

[15] Sejersted O. M., Sjogaard G. (2000). Dynamics and consequences of potassium shifts in skeletal muscle and heart during exercise. *Physiological Reviews*.

[16] Clausen T. (2003). Na^+^-K^+^ pump regulation and skeletal muscle contractility. *Physiological Reviews*.

[17] Clausen T. (2013). Quantification of Na^+^,K^+^ pumps and their transport rate in skeletal muscle: functional significance. *Journal of General Physiology*.

[18] Orlowski J., Lingrel J. B. (1988). Tissue-specific and developmental regulation of rat Na,K-ATPase catalytic *α* isoform and *β* subunit mRNAs. *The Journal of Biological Chemistry*.

[19] Yuan X., Lin Z., Luo S., Ji G., Yuan C., Wu Y. (2007). Effects of different magnitudes of cyclic stretch on Na^+^-K^+^-ATPase in skeletal muscle cells *in vitro*. *Journal of Cellular Physiology*.

[20] Kristensen M., Rasmussen M. K., Juel C. (2008). Na^+^-K^+^ pump location and translocation during muscle contraction in rat skeletal muscle. *European Journal of Physiology*.

[21] Murphy K. T., Nielsen O. B., Clausen T. (2008). Analysis of exercise-induced Na^+^-K^+^ exchange in rat skeletal muscle *in vivo*. *Experimental Physiology*.

[22] Juel C. (2009). Na^+^-K^+^-ATPase in rat skeletal muscle: Muscle fiber-specific differences in exercise-induced changes in ion affinity and maximal activity. *The American Journal of Physiology - Regulatory Integrative and Comparative Physiology*.

[23] Nordsborg N. B., Kusuhara K., Hellsten Y. (2010). Contraction-induced changes in skeletal muscle Na^+^,K^+^ pump mRNA expression—importance of exercise intensity and Ca^2+^-mediated signalling. *Acta Physiologica*.

[24] Krivoi I. I., Drabkina T. M., Kravtsova V. V. (2006). On the functional interaction between nicotinic acetylcholine receptor and Na^+^,K^+^-ATPase. *Pflügers Archiv*.

[25] Heiny J. A., Kravtsova V. V., Mandel F. (2010). The nicotinic acetylcholine receptor and the Na,K-ATPase *α*2 isoform interact to regulate membrane electrogenesis in skeletal muscle. *The Journal of Biological Chemistry*.

[26] Krivoi I., Vasiliev A., Kravtsova V., Dobretsov M., Mandel F. (2003). Porcine kidney extract contains factor(s) that inhibit the ouabain-sensitive isoform of Na,K-ATPase (*α*2) in rat skeletal muscle: a convenient electrophysiological assay. *Annals of the New York Academy of Sciences*.

[27] Chibalin A. V., Heiny J. A., Benziane B. (2012). Chronic nicotine modifies skeletal muscle Na,K-ATPase activity through its interaction with the nicotinic acetylcholine receptor and phospholemman. *PLoS ONE*.

[28] Radzyukevich T. L., Neumann J. C., Rindler T. N. (2013). Tissue-specific role of the Na,K-ATPase *α*2 isozyme in skeletal muscle. *The Journal of Biological Chemistry*.

[29] Pierno S., Desaphy J.-F., Liantonio A. (2007). Disuse of rat muscle in vivo reduces protein kinase C activity controlling the sarcolemma chloride conductance. *The Journal of Physiology*.

[30] Williams M. W., Resneck W. G., Kaysser T. (2001). Na,K-ATPase in skeletal muscle: Two populations of *β*-spectrin control localization in the sarcolemma but not partitioning between the sarcolemma and the transverse tubules. *Journal of Cell Science*.

[31] Murphy K. T., Macdonald W. A., McKenna M. J., Clausen T. (2006). Ionic mechanisms of excitation-induced regulation of Na^+^-K^+^-ATPase mRNA expression in isolated rat EDL muscle. *American Journal of Physiology—Regulatory Integrative and Comparative Physiology*.

[32] Rasmussen M. K., Kristensen M., Juel C. (2008). Exercise-induced regulation of phospholemman (FXYD1) in rat skeletal muscle: implications for Na^+^/K^+^-ATPase activity. *Acta Physiologica*.

[33] Benziane B., Björnholm M., Pirkmajer S. (2012). Activation of AMP-activated protein kinase stimulates Na^+^,K^+^-ATPase activity in skeletal muscle cells. *The Journal of Biological Chemistry*.

[34] Juel C. (2014). Oxidative stress (glutathionylation) and Na,K-ATPase activity in rat skeletal muscle. *PLoS ONE*.

[35] He S., Shelly D. A., Moseley A. E. (2001). The *α*1- and *α*2-isoforms of Na-K-ATPase play different roles in skeletal muscle contractility. *The American Journal of Physiology—Regulatory Integrative and Comparative Physiology*.

[36] Radzyukevich T. L., Moseley A. E., Shelly D. A. (2004). The Na^+^-K^+^-ATPase *α*
_2_-subunit isoform modulates contractility in the perinatal mouse diaphragm. *American Journal of Physiology: Cell Physiology*.

[37] Radzyukevich T. L., Lingrel J. B., Heiny J. A. (2009). The cardiac glycoside binding site on the Na,K-ATPase *α*2 isoform plays a role in the dynamic regulation of active transport in skeletal muscle. *Proceedings of the National Academy of Sciences of the United States of America*.

[38] Thomason D. B., Booth F. W. (1990). Atrophy of the soleus muscle by hindlimb unweighting. *Journal of Applied Physiology*.

[39] Ogneva I. V., Biryukov N. S., Leinsoo T. A., Larina I. M. (2014). Possible role of non-muscle alpha-actinins in muscle cell mechanosensitivity. *PLoS ONE*.

[40] Thompson C. B., Dorup I., Ahn J., Leong P. K. K., Mcdonough A. A. (2001). Glucocorticoids increase sodium pump *α*
_2_- and *β*
_1_-subunit abundance and mRNA in rat skeletal muscle. *American Journal of Physiology: Cell Physiology*.

[41] Petersen A. C., Murphy K. T., Snow R. J. (2005). Depressed Na^+^-K^+^-ATPase activity in skeletal muscle at fatigue is correlated with increased Na^+^-K^+^-ATPase mRNA expression following intense exercise. *American Journal of Physiology—Regulatory Integrative and Comparative Physiology*.

[42] Ohira M., Hanada H., Kawano F., Ishihara A., Nonaka I., Ohira Y. (2002). Regulation of the properties of rat hind limb muscles following gravitational unloading. *Japanese Journal of Physiology*.

[43] De-Doncker L., Kasri M., Picquet F., Falempin M. (2005). Physiologically adaptive changes of the L_5_ afferent neurogram and of the rat soleus EMG activity during 14 days of hindlimb unloading and recovery. *Journal of Experimental Biology*.

[44] Walaas S. I., Czernik A. J., Olstad O. K., Sletten K., Walaas O. (1994). Protein kinase C and cyclic AMP-dependent protein kinase phosphorylate phospholemman, an insulin and adrenaline-regulated membrane phosphoprotein, at specific sites in the the carboxy terminal domain. *Biochemical Journal*.

[45] Mahmmoud Y. A., Cornelius F. (2002). Protein kinase C phosphorylation of purified Na,K-ATPase: c-terminal phosphorylation sites at the *α*- and *γ*-subunits close to the inner face of the plasma membrane. *Biophysical Journal*.

[46] Berchtold M. W., Brinkmeier H., Müntener M. (2000). Calcium ion in skeletal muscle: its crucial role for muscle function, plasticity, and disease. *Physiological Reviews*.

[47] Ingalls C. P., Wenke J. C., Armstrong R. B. (2001). Time course changes in [Ca^2+^]_i_, force, and protein content in hindlimb-suspended mouse soleus muscles. *Aviation, Space, and Environmental Medicine*.

[48] Xu P.-T., Li Q., Sheng J.-J., Chang H., Song Z., Yu Z.-B. (2012). Passive stretch reduces calpain activity through nitric oxide pathway in unloaded soleus muscles. *Molecular and Cellular Biochemistry*.

[49] Blaustein M. P., Golovina V. A. (2001). Structural complexity and functional diversity of endoplasmic reticulum Ca^2+^ stores. *Trends in Neurosciences*.

